# Training and support for dementia caregivers in the Middle East and North Africa region: a scoping review

**DOI:** 10.3389/fpubh.2026.1719399

**Published:** 2026-02-27

**Authors:** Jackie Hoi Man Chan, Alia D. Alhaddad, Jacqueline Maria Dias

**Affiliations:** 1 Department of Nursing, Faculty of Health Sciences, Higher Colleges of Technology, Dubai, United Arab Emirates; 2 Division of Nursing, School of Humanity, Social Sciences & Health, University of Wollongong in Dubai, Dubai, United Arab Emirates; 3 Sheikh Khalife General Hospital, Umm Al Quwain, United Arab Emirates; 4 Department of Nursing, College of Health Sciences, University of Sharjah, Sharjah, United Arab Emirates

**Keywords:** caregivers, dementia, Middle East, North Africa, support, training, review

## Abstract

**Introduction:**

The anticipated rise in dementia cases across the Middle East and North Africa (MENA) region, particularly the staggering 1795% projected increase in the United Arab Emirates by 2050, underscores an urgent need for community-based training for informal caregivers and professional training for formal caregivers. This scoping review mapped the evidence on dementia caregiving training for informal and formal caregivers in this region.

**Methods:**

The Joanna Briggs Institute methodology for scoping reviews was adopted. Four electronic databases were searched from inception to April 2025. Two authors independently screened and extracted data. The PAGER framework was employed to collate and critique the findings, identify advances and gaps, discuss evidence for practice, and suggest recommendations for practice and research.

**Results:**

Ten included studies reported training for informal caregivers, including educational programs (*n* = 5) and psychosocial trainings (*n* = 7) (i.e., psychoeducation, counselling and psychotherapy, multicomponent interventions, and miscellaneous interventions). Only two included studies reported training (i.e., educational program) for formal caregivers. There appeared to be a scarcity of evidence on dementia caregiving training for informal and formal caregivers in the MENA region, and the training was skewed toward educational programs. The evaluations focused on a deficit-based perspective (i.e., burden and stress, depressive symptoms, and anxiety) that primarily focused on the caregivers.

**Discussion:**

The findings provided insights into dementia caregiving training for informal and formal caregivers in the MENA region. Further research on systematic reviews to evaluate overall training effectiveness is warranted. Additionally, studies evaluating culturally relevant psychosocial interventions, particularly those focus on strength-based outcomes (e.g., positive aspects of caregiving, caregiving confidence, and social support) to inform practice, are highly recommended. Furthermore, validation of region-specific measurement tools should be a priority.

**Systematic review registration:**

https://doi.org/10.17605/OSF.IO/UA9E3

## Introduction

Dementia, a non-communicable disease, is currently the 7th leading cause of death worldwide (1). Alzheimer’s Disease International forecasted that dementia will be the 3rd leading NCD cause of death globally by 2040 (2). It is projected that the prevalence of dementia in the Middle East and North Africa (MENA) region will increase by 367% by 2050 (3). Notably, Qatar will have the largest increase (1926%, 85,046 cases), followed by the United Arab Emirates (1795%, 221,672 cases) and Bahrain (1,084%, 60,650 cases) (3). Dementia, therefore, has emerged as a pressing public health issue in the MENA region, necessitating utmost attention to understand the situation. Although new medical treatment (i.e., amyloid-targeting therapies, ATTs) for Alzheimer’s disease (AD) is now available, it is limited to the early stage of the disease, to some areas (e.g., UAE) in the MENA region (4), and by the high cost (approximately USD 26500 per year) (5). These limitations may imply that dementia caregiving provided by informal and formal caregivers should not be overlooked in the management of dementia. Indeed, the latest consensus among the leading clinical neurologists suggests that patient and caregiver education be part of the management of Alzheimer’s disease in the UAE (4). Meanwhile, evidence suggests that specialized dementia caregiving, such as person-centered care, demonstrated a moderate effective size in reducing behavioral and psychological symptoms in dementia (BPSD) and improving cognitive function in people living with dementia (PLWD) (6). It is therefore worthwhile to understand the training and support available for dementia caregiving in the local context.

### Disease trajectory

Dementia is a syndrome characterized by the progressive destruction of neurons in the brain, primarily caused by neuroinflammation in AD (7). Such neuroinflammation is triggered by deposition of beta-amyloid and tau, and is fueled by factors such as cell debris and pro-inflammatory factors (e.g., cytokines), creating a cycle of neuronal damage and dysfunction. Consequently, progressive cognitive and functional decline occurs (4). Cognitive decline leads to varying degrees of cognitive impairment, including difficulties in communication, executive dysfunction, and behavioral manifestations of distress (e.g., hallucinations, agitation) (1). Therefore, the ability to perform instrumental activities of daily living, such as managing household chores and finances, is significantly impacted (8). Meanwhile, functional decline further contributes to challenges with activities of daily living, such as feeding and walking (8). This combination of cognitive and functional decline impairs the ability of PLWD to function in daily life and eventually leads to a high dependency on caregiving.

The latest consensus on management of AD in the UAE suggests early detection and prevention through lifestyle modification to promote brain health (4). It also suggests ATTs for eligible patients in the early stage. In moderate-to-severe AD, a patient-centered approach through symptom management is recommended to optimize quality of life. In late-stage AD, management should focus on compassionate care, dignity, and supporting families. Although ATTs are now available, they are currently limited to the early stages of the disease and to specific geographic locations (e.g., the UAE) (4). Therefore, dementia caregiving, particularly patient-centered care, by both informal and formal caregivers, continues to play a significant role throughout disease progression, including the onset of neuropsychiatric symptoms and the maintenance of cognitive function (6, 9).

### Global dementia caregiving trends

Worldwide, 80% of PLWD live at home and are cared for by informal caregivers (i.e., family members, relatives, or friends who are not paid); the remaining 20% may be cared for by privately hired formal caregivers or reside in long-term care facilities (1). Competency in dementia caregiving among both informal and formal caregivers is therefore critical to provide high-quality care. Notably, person-centered care, which takes into account the psycho-social-cultural perspectives, is commonly employed to maintain the physical and mental well-being of the PLWD (10). WHO (1) states that relevant education and support for both informal and formal caregivers are necessary to provide such care. For informal caregivers, several meta-analyses suggested that psychosocial training was promising in enhancing caregiving self-efficacy, knowledge and ability, as well as their mental health (11, 12). Additionally, improvements in physical health and neuropsychiatric symptoms of PLWD were demonstrated. Examples of psychosocial training include psychoeducation, counselling and psychotherapy, support groups, mindfulness-based interventions, and multicomponent interventions (13). For formal caregivers, educational programs, particularly those involving communication skills, person-centered care, and dementia-care mapping with supervision, demonstrated positive outcomes on agitation and cognitive function in PLWD, according to meta-reviews (6, 9).

### Dementia caregiving in the MENA region

Both informal and formal caregivers play important roles in dementia caregiving in the MENA region, given the region’s unique culture and healthcare system dynamics. PLWD are predominantly cared for at home in the MENA region due to Arab Islamic culture, in which multigenerational households are common, fostering strong family ties and high respect for seniors; only a small proportion reside in long-term care facilities (14). Caregiving, therefore, falls to family members, particularly women (i.e., mothers, wives, daughters) (14). The literature suggests that informal caregivers encounter difficulties and significant adjustments in relationships and expectations when they transition into a caregiving role (15). However, most informal caregivers in this region have reported a lack of knowledge and skills related to dementia caregiving (14). Very often, these unprepared informal caregivers report high levels of caregiving burdens, depression, and anxiety (16). These negative consequences of caregiving could lead to neglect and avoidance in the informal caregivers and thereby result in decreased quality of life in PLWD, frequent hospitalization, exacerbated BPSD, or physical abuse (17).

In some high-income areas in the MENA region, formal caregivers, such as domestic helpers, personal care aides, home health aides, and professional caregivers (e.g., nurses and nursing assistants), are commonly hired to provide the caregiving for those PLWD who reside at home (18, 19). They provide direct care, including basic and instrumental activities of daily living. However, Bar-Tur (18) suggested that formal caregivers, such as domestic helpers, may not provide dementia specific care without relevant training, worsening the neuropsychiatric symptoms, such as delusions of the PLWD.

Professional caregivers in long-term care facilities or clinical settings also have reported a lack of preparedness in dementia caregiving in the MENA region (20). A cross-sectional survey in Qatar found that healthcare professionals (i.e., physicians, nurses, and medical students) had a moderate level of dementia knowledge and were particularly lacking in knowledge of recent advances in dementia pathophysiology (21). Another survey of Egyptian medical students found that only half fully understood dementia caregiving (22). Healthcare staff having inadequate training on dementia caregiving resulted in poor communication skills, negative attitudes, and low caregiving confidence, affecting the quality of care, and in turn affected the quality of life of PLWD and their disease progression (23–25). Notably, inadequate knowledge of dementia care among healthcare professionals in clinical settings was associated with higher adverse post-operative outcomes, including respiratory problems, urinary tract infections, and use of restraints, in PLWD (26).

Relevant training, such as basic disease knowledge, daily caregiving skills, communication techniques, managing challenging behaviors, and stress coping, is therefore necessary to prepare both informal and formal caregivers to meet the individual care needs of PLWD, while addressing their own mental well-being (10). Without a commitment to preparing the caregivers, the ever-changing and complex needs associated with the progression of the disease may not be fully addressed, impacting not only the quality of life of the PLWD but also the caregivers.

### Gaps in caregiving training in the MENA region

Nevertheless, a scarcity of evidence on training and support for both informal and formal caregivers of PLWD has been reported in the MENA region (20, 27–30). According to a systematic review of psychosocial training for informal caregivers, only 4 of 131 (3%) MENA-based randomized controlled trials were included; the rest were conducted in North America, Europe, and Asia (11). Notably, contextually and culturally tailored training and support for caregivers are essential for providing dementia care that is relevant to Arab-Islamic culture in the MENA region. To the author’s knowledge, no review systematically maps the evidence of training and support for both informal and formal caregivers of PLWD in the MENA region. This represents a critical gap in providing evidence to improve the quality of life and slow disease progression among PLWD in this region. Aligning with the latest consensus on the management of Alzheimer’s disease in the UAE (4), mapping the training and support available for dementia caregiving for informal caregivers in the MENA region can help tailor family caregiving training to Arab-Islamic cultures. Similarly, such evidence for formal caregivers can help prepare the healthcare workforce to provide high-quality and culturally relevant dementia care in the MENA region.

This scoping review aimed to systematically identify and map the training and support available for informal and formal dementia caregivers in the MENA region. The objectives included: (1) identifying the training for informal caregivers of people living with dementia who live in the community and the respective study outcomes, (2) identifying the training for formal caregivers of people living with dementia and the respective study outcomes.

## Methods

The Joanna Briggs Institute (JBI) methodology for scoping reviews (31) was adopted to conduct this review. The nine-stage framework includes (1) defining and aligning the objectives and questions; (2) developing and aligning the inclusion criteria with objectives and questions; (3) describing the planned approach to evidence searching, selection, data extraction, and presentation of the evidence; (4) searching for the evidence; (5) selecting the evidence; (6) extracting the evidence; (7) analysing the evidence; (8) presenting the results; and (9) summarizing the evidence in relation to the review’s purpose, making conclusions, and noting any implications of the findings. In particular, the PAGER framework was used to systematically synthesize results to maximize the utilization of this report by healthcare practitioners, nurse educators, and researchers (32). Details of PAGER framework were provided in “Evidence analysis” in the following sections. This scoping review did not involve human participants; therefore, ethical approval was not required. Additionally, since the nature of scoping review is to map the landscape of evidence but not evaluate the effectiveness, assessment of methodological bias is not required according to the JBCI guideline for scoping review (31). This review followed the guidelines of the Preferred Reporting Items for Systematic Reviews and Meta-Analysis Extension for Scoping Reviews (PRISMA-ScR) (33). This review was prospectively registered at Open Science Framework on 4 September 2025 (https://doi.org/10.17605/OSF.IO/UA9E3).

### Eligibility criteria

The Population, Concept, and Context framework was employed to determine which articles were eligible for inclusion according to the JBI guidelines (31) (Table 1).

**Table 1:** Eligibility criteria.

Framework	Keywords	Inclusion criteria	Exclusion criteria
Population	Caregivers of people with dementia: informal caregiver, formal caregiver, nurses, family	Informal caregivers are the family members or friends who provide caregiving without paid Formal/professional caregivers are those received medical training such as nurses	–
Context	Middle East, North Africa, UAE, Egypt, Jordan, Israel, Iran, Turkey	The Middle East and North Africa region according to the WHO	–
Concept	Training, support, intervention, program	Psychosocial training and support that help equip caregivers with caregiving knowledge and skills, and provide emotional support during dementia caregiving. Examples include but not limited to psychoeducation and psychotherapy	–
Study design	–	Quantitative study, qualitative study, mixed-methods study	Reviews, editorials, commentary articles, protocol, conference abstract, case studies or review

#### Inclusion criteria

The target population was the caregivers of people living with dementia at any stage, who may be informal or formal caregivers. Informal caregivers are the family members or friends who provide care without being paid (43). Formal caregivers are those who are paid to provide care (24). Examples include, but are not limited to, domestic helpers and healthcare professionals, such as private nurses.

The concept in this study was any training or support for informal and formal caregivers that helps equip them with the necessary dementia caregiving knowledge and skills or provides emotional support during caregiving in the community. Examples included, but were not limited to, educational programs and psychosocial training, such as psychoeducation and psychotherapy.

The context was the 19 countries in the Middle East and North Africa region, as reported by the United Nations (34). Primary research studies, including those with quantitative, mixed-methods, and qualitative designs, were included. Only articles that were published in English were included.

#### Exclusion criteria

Editorial, letters, commentary, conference abstracts, protocol, conference abstracts, case studies, and reviews were excluded.

### Search strategy

A literature search was done from inception to April 2025. A three-step search strategy was adopted, including: (1) identifying key words by an initial limited search of a databases (PubMed); (2) searching all included databases (PubMed, Scopus, CINAHL and Google Scholar) by using the identified keywords ((“informal caregiver” OR “formal caregiver” OR “nurses” OR “family”) AND (“Middle East” OR “North Africa” OR “UAE” OR “Egypt” OR “Jordan” OR “Israel” OR “Iran” OR “Turkey”) AND (“Training” OR “support” OR “intervention” OR “program”)). These databases are commonly adopted to identify high-quality and peer-reviewed publications in the relevant areas, and (3) manually searching the reference list of identified reports and articles. A combination of keywords in either title or abstract was used. The search was limited to English-language articles. All articles were exported to EndNote 20. Duplicate articles were removed. A PRISMA flow diagram was used to illustrate the selection process for the articles.

### Data extraction

The data extraction form was developed in accordance with the review objectives. The extracted data included: (1) characteristics of the studies—including authors, year of publication, sample size, and study design, (2) training for informal caregivers that are currently available in the MENA region, (3) training for formal caregivers that are currently available in the MENA region, (4) outcome variables and the measurement tool for the caregivers in the identified training. All data extracted were presented in Table 2. Two researchers (JC and JD) independently extracted data from the eligible studies. The extracted data were compared, and any discrepancies were resolved through discussion among the authors until a consensus was reached.

**Table 2:** Characteristics of the included studies and the identified interventions.

References	Region	Sample size	Study design	Participants characteristics	Intervention	Main findings
Training for informal caregivers						
Abdelhalimet et al. (43)	Egypt	84 (intervention = 42; control = 42)	RCT	Informal caregiver Median age range: 39.2–40.5 Gender: Female 73.8% Relationship with CR: Daughter 59.5% Education level: Secondary or above 70.2% Caregiving experience: not reported Care recipients’ dementia stage: Mild to moderate stage	Psychoeducation, skill-based 6 biweekly sessions, 1.5-2 h each Major topics: basic dementia knowledge, communication skills, management of disruptive behaviors	Caregiver burden Measuring tool: Zarit Burden Interview Results: Significant reduction Anxiety Measuring tool: 7-item Generalized Anxiety Disorder Scale Result: Significant reduction Neuropsychiatric symptoms Measuring tool: Neuropsychiatric Inventory Questionnaire Result: No effect
Heydari et al. (38)	Iran	72 (intervention = 36; control = 36)	RCT	Informal caregivers Mean age: 49.7 Gender: Female 65.2% Relationship with CR: Not reported Education level: Diploma or above 38.8% Caregiving experience: Not reported Care recipients’ dementia stage: Intense Alzheimer 55%	Educational program, focused on problem-oriented coping strategies training 8 weekly sessions, 45 min each Major topics: coping strategies building on problem-solving, anger and stress management, coping with negative affect and cognitive deterioration, relationship-maintenance strategies	Quality of life Measuring tool: 36-item Short Form Health Survey (SF-36), Iranian version Results: Significant improvement in subscale: general health perception, role limitations due to physical problems, role limitations due to emotional problems, social function, vitality and mental health
Jahani et al. (28)	Iran	70 (intervention = 35; control = 35)	RCT	Informal caregiver Mean age: 44.4 Gender: Female 82.8% Relationship with CR: Not reported Education level: Secondary or above 100% Caregiving experience: 2.5–2.6 years Care recipients’ dementia stage: Mild to moderate stage	Psychological intervention, compassion-based 5 weekly sessions Major topics: principles of compassion, compassion skill, mindfulness training	Caregivers’ grief Measuring tool: Caregiver Grief Inventory Result: Significant reduction immediate post- and one-month post-intervention
Javadpour et al. (42)	Iran	29	One group pre-post-test	Informal caregiver Mean age: 49.0 Gender: Female 100% Relationship with CR: Adult Child 37.9% Education level: Not reported Caregiving experience: not reported Care recipients’ dementia stage: not reported	Educational program 8 weekly sessions, 1.5 h each Major topics: basic dementia knowledge, management of disruptive behaviors, discussion on caregiving experience among group	Caregiver stress Measuring tool: 10-item Perceived Stress Scale Result: Significant reduction Perceived health General Health Questionnaire Result: Significant reduction Neuropsychiatric symptoms Measuring tool: Classic Neuropsychiatry Inventory Result: Significant reduction
Kuzu et al. (36)	Turkey	32 pairs of dyads	One group pre-post-test	Informal caregivers Mean age: 49.6 Gender: Female 65.6% Relationship with CR: Not reported Education level: Secondary 50% Caregiving experience: not reported Care recipients’ dementia stage: not reported Care recipients’ mean Mini Mental State Examination score: 11.6	Educational program, ‘Comprehensive Educational Program Reinforced by an Individualized Component’ (CEPRIC) 1 session, 50 min Major topics: basic dementia knowledge, management of problems defined by nursing diagnoses, educational booklet	Depressive symptoms Measuring tool: Beck Depression Inventory Result: Significant reduction Anxiety Measuring tool: Beck Anxiety Inventory Result: Significant reduction Quality of life Measuring tool: Duke Scale Result: Significant increase in physical health and general health Problems defined by Nursing Diagnoses Guideline under NANDA Results: Significant reduction in impaired social interaction, caregiver role strain, ineffective individual coping, ineffective family coping; Significant enhancement in the care recipient: Altered sensory-perceptual processes, alternation in nutrition, powerlessness, disturbed sleep pattern, risk for trauma, anxiety, self-care deficit
Mahdavi et al. (39)	Iran	100 (spiritual therapy = 33; group session control = 32; control = 35)	RCT	Informal caregivers Mean age: 52.9 Gender: Female (percentage not reported) Relationship with CR: Adult Child (45%); Spouse (32%); In-laws (6%); Grandchild (6%) Education level: Secondary or above 57% Caregiving experience: not reported Care recipients’ dementia stage: Not reported Care recipients’ mean Mini-Mental State Examination score: Not reported	Spiritual group therapy based on Iranian and Islamic culture 5 weekly sessions, 45–60 min each Including prayers, discussions about divine issues, and using holy books for treatment, illustration and relaxation skills Covering group solidarity, role of reciting the Quran and prayers, and the experience of spiritual care and its effects	Caregiver strain Measuring tool: Robinson’s Caregiver Strain Index Results: Significant reduction in caregiver strain in the spiritual therapy group
Pahlavanzadeh et al. (37)	Iran	60 pairs of dyads (intervention = 30; control = 30)	RCT	Informal caregiver Informal caregivers Mean age: 44.7 Gender: Female 76% Relationship with CR: Adult Child (76%); Spouse (38%) Education level: Diploma or above 32% Caregiving experience: 2.2 years Care recipients’ dementia stage: Not reported Care recipients’ mean Mini-Mental State Examination score: 13.7	Family education program 5 weekly group sessions, 90 min each Major topics: basic dementia knowledge, communication skills, and techniques to take care of care recipient	Caregiver burden Measuring tool: Zarit Caregiver Burden Scale Result: Significant reduction in caregiver burden
Salamizadeh et al. (40)	Iran	60 (intervention = 30; control = 30)	RCT	Informal caregiver Mean age: 48.9 Gender: Female 76.6% Relationship with CR: Adult Child 53.3% Education level: Secondary or above 55% Caregiving experience: ≥ 6 months Care recipients’ dementia stage: not reported	Educational program, spiritual care based 5 weekly sessions, 45–60 min each Major topics: seeking help from holy people, patience, generosity, altruism, mantra, and prayer	Self-efficacy Measuring tool: 10-item General Self-Efficacy Scale Result: Significant improvement
Shata et al. (41)	Egypt	120 (intervention = 60; control = 60)	RCT	Informal caregiver Mean age: 69.2 Gender: Female 67.5% Relationship with CR: Adult Child 45.6% Education level: Secondary 44.7% Caregiving experience: not reported Care recipients’ dementia stage: not reported	Multicomponent program 8 weekly sessions, 45–60 min each Components: psychoeducation, cognitive behavioral therapy, support group Major topics: basic dementia knowledge, management of problem behaviors, stress coping Cultural sensitive session (details not reported)	Dementia knowledge Measuring tool: Alzheimer’s Disease Knowledge Test Result: Significant improvement immediate post-intervention and at 3-month follow up Depressive symptoms Measuring tool: Hamilton Depression Rating Scale Result: Significant improvement immediate post-intervention and at 3-month follow up Anxiety Measuring tool: Taylor Manifest Anxiety Scale Result: Significant improvement immediate post-intervention and at 3-month follow up Caregiver burden Measuring tool: Zarit Burden Interview Result: Significant improvement immediate post-intervention and at 3-month follow up
Tawfik et al. (29)	Egypt	68 (intervention = 34; control = 34)	RCT	Informal caregiver Mean age: 37.8 Gender: Female 80% Relationship with CR: Daughters 41.6% Education level: Secondary 38.3% Caregiving experience: not reported Care recipients’ dementia stage: not reported	Psychoeducation 8 weekly sessions, 1 h each Major topics: basic dementia knowledge, management of problem behaviors, stress coping	Caregiver burden Measuring tool: Zarit Burden Interview Result: Significant improvement Quality of life Measuring tool: Quality of Life in Alzheimer Disease Result: Significant improvement
Werner et al.	Israel	100 (intervention = 54; control = 46)	RCT	Informal caregiver Mean age: 73.4 Gender: Female 69% Relationship with CR: Spouse 100% Education level: Secondary or above 85% Caregiving experience: not reported Care recipients’ dementia stage: Mild to moderate 99.9%	Counselling, “New York University Caregiver Intervention” 6 counselling: 2 with the caregivers, and 4 with the caregivers and the family members together, *ad hoc* counselling to manage crisis and care recipients’ symptoms	Depressive symptoms Measuring tool: Geriatric Depression Scale Result: Insignificant reduction in group × time effect; only significant reduction in group effect
Zamani-Alavijeh et al. (15)	Iran	38	One group pre-post-test	Informal caregiver Mean age: 40.9 Gender: Female 73.7% Relationship with CR: Not reported Education level: Secondary or above 68.4% Caregiving experience: ≥ 6 months Care recipients’ dementia stage: Not reported	Educational program, guided by the Progressively Lowered Stress Threshold extended model 8 sessions, 2 h each Major topics: basic dementia knowledge, daily caregiving, communication skill, management of problem behaviors, self-efficacy enhancement Culture-specific strategies Play Quran recitation at the specific times of the day Preform prayers when the care recipient is present Do not argue with the care recipient regarding the time, place, and the sequence of performing prayers or any religious rituals Put henna on a female patient Attend poetry nights, especially for male patients	Dementia knowledge Measuring tool: Self-developed questionnaire, 30 items Result: Significant enhancement Practice and behaviors of caregiving Measuring tool: Self-developed questionnaire, 40 items Result: Significant enhancement
Training for formal caregivers						
Hayajneh and Shehadeh (44)	Jordon	15	Mixed methods	Formal caregiver (long-term care facilities setting) Age range: 25–47 Gender: Female 70% Caregiving experience: 2–9 years Professions: Nurses, general physician, psychiatrist, physiotherapist, dietitian	Educational program “See the person first,” focused on person-centered care 4 weeks: 10-h theoretical lectures, 20-h clinical training Major topics: basic dementia knowledge, person-centered care skills	Caregivers’ person-centered behavior Measuring tool: Global Behavior Scale questionnaire Result: lowest post-test score was 73/77 Semi-structured interview Three themes: Acceptance, Empathy, and Resiliency
Rezq & Gutierrez (20)	Saudi Arabia	50	One group pre-post-test	Formal caregiver (long-term care facilities setting) Mean age: 34.3 Gender: Female 78% Relationship with CR: Adult Child 45.6% Education level: Secondary 44.7% Caregiving experience: 6.2 years Education level: Nursing diploma 12%; non-nursing diploma 20%	Educational program Number of sessions and frequencies not reported, each session 30 min Major topics: basic dementia knowledge, caregiving skill (e.g., toileting, grooming), communication skill	Caregiver burden Measuring tool: Self-developed questionnaire Result: Significant reduction immediate post-intervention and at 3-month follow-up
Note. CR: care recipient; NANDA: North American Nursing Diagnosis Association; RCT: randomized controlled trial						

### Evidence analysis

The PAGER framework (32) was employed to comprehensively describe and critique the findings of this scoping review. The findings were analyzed, presented, and summarized across five aspects: (i) patterns, (ii) advances, (iii) gaps, (iv) evidence for the practice, and (v) research recommendations. Key themes were structured as patterns according to the research question. For each pattern, advancements in the field (e.g., theoretical and methodological), and any identified knowledge or practice gaps were described. Based on the information regarding patterns, advances, and gaps, contextualized evidence for practice and research recommendations were provided. The analysis using the PAGER framework is presented in Table 3.

**Table 3:** PAGER analysis.

Themes	Advances	Gaps	Evidence for practice	Research recommendations
Training for informal caregivers and the study outcomes in the MENA region	There is growing evidence of community-based training for informal caregivers in the MENA region since 2005	There is a paucity of evidence in psychosocial trainings, particularly mindfulness-based interventions, support groups, care coordination and case management, and training of the care recipient with caregiver involvement There is a paucity of evidence in the psychosocial outcomes regarding social support and positive aspects of caregiving, and the outcomes primarily focused on the family caregivers’ mental health There is a paucity of information regarding culturally sensitive strategies in caregiver training There is a paucity of qualitative evidence on community-based training	There is growing evidence of community-based educational programs and psychosocial interventions (i.e., psychoeducation, counselling and psychotherapy, multicomponent interventions, miscellaneous interventions) in the mental health of informal caregivers and caregiving knowledge and skill	More research is needed in various psychosocial interventions, and qualitative design in evaluating psychosocial outcomes on both family caregivers and care recipients
Training for formal caregivers and the study outcomes in the MENA region	There is growing evidence of educational programs for formal caregivers in the MENA region since 2014	There is a paucity of evidence in psychosocial interventions in general There is a paucity of evidence in the psychosocial outcomes regarding social support and positive aspects of caregiving, and the outcomes on the PLWD (e.g., frequency of neuropsychiatric symptoms, occurrence of disruptive behaviors) There is a paucity of information regarding culturally sensitive strategies in caregiver training	Evidence to emerge from future research on the training for formal caregivers in the MENA region	More interventional research in general is necessary to provide evidence on training effectiveness Large scale studies with rigorous designs and qualitative designs are warranted to understand comprehensively the dementia caregiving among informal caregivers and their care recipients

MENA, Middle East and North Africa; PLWD, people living with dementia.

## Results

### Search results

**Figure 1:**
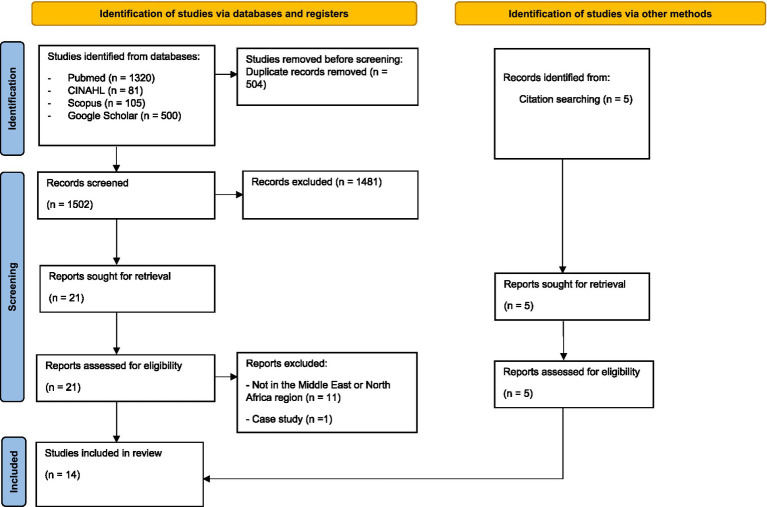
PRISMA flow diagram.

We identified 2,006 records from four databases, of which 504 were duplicates. Five studies were identified through a manual search of the reference lists of relevant articles. After screening the titles and abstracts, 1,481 records were excluded, and 21 records were selected for full-text screening. Eventually, 14 articles were included for data extraction. Figure 1 shows the PRISMA flow diagram of the study selection process. Owing to the inductive nature of scoping reviews, an assessment of methodological limitations or risk of bias in the evidence was not required (31, 35). The results were divided into five sections: (1) study characteristics, (2) psychosocial training for informal caregivers and the respective study outcomes, (3) psychosocial training for informal caregivers and the respective study outcomes.

### Study characteristics

The 14 articles included in this review were published between 2005 and 2025, with a surge of 12 publications since 2010. The 14 included articles were one-group post-test studies (*n* = 4), randomized controlled trials (*n* = 9), and mixed-methods studies (*n* = 1). The study sites included Iran (*n* = 7), Egypt (*n* = 3), Turkey (*n* = 1), Jordon (*n* = 1), Israel (*n* = 1), and Saudi Arabia (*n* = 1). Twelve articles were related to informal caregivers, and two were related to formal caregivers. Two articles involved dyads (36, 37), and the rest involved only caregivers. The sample sizes of the included studies ranged from 10 to 120, totalling 893 participants in this review. Table 2 summarizes the study characteristics. Table 3 summarizes the analyses based on the PAGER framework. The following narrative synthesis presented the results of the scoping review, which was facilitated by the PAGER framework.

### Training for informal caregivers and the study outcomes

There were 12 included articles reporting training for informal caregivers (i.e., family members) since 2005, with a surge of 10 articles since 2010. Study designs were dichotomised as RCT (*n* = 9) (27–30, 37–41) and one-group pre-posttest (*n* = 3) (15, 36, 42). It appears that there is a gap in qualitative design to explore the experience of informal caregivers in the training program.

Two primary training programs were identified: educational programs (*n* = 5), and psychosocial trainings (*n* = 7). Education programs provided a didactic component only, and they were further categorized as general and theme-based. General educational programs provided a basis for dementia disease knowledge, daily caregiving skills, communication skills, and management of BPSD (15, 36, 37, 42). In addition to these common caregiving topics, Heydari et al. (38) focused on problem-oriented coping strategies and Salamizadeh et al. (40) focused on spiritual care.

For psychosocial training, four types were classified according to Cheng et al. (11): psychoeducation (*n* = 2), counselling and psychotherapy (*n* = 2), multicomponent interventions (*n* = 1), and miscellaneous interventions (*n* = 1). Psychoeducation, characterized by a psychotherapeutic technique such as cognitive-behavioral technique, primarily covered the basic dementia caregiving knowledge and skill, and stress coping for the informal caregivers (29, 43). Compassion-based psychological intervention (28) and counselling (30) were identified under the category of counselling and psychotherapy. Shata et al. (41) employed a multicomponent intervention that included psychoeducation, cognitive behavioral therapy, and a support group. Mahdavi et al. (39) employed spiritual group therapy, which involved prayers, discussions about divine issues, and the use of holy books for treatment and illustration.

Evidence on training and support available for informal caregivers in the MENA region appears to skew toward educational programs. There is a lack of evidence on psychosocial trainings, including mindfulness-based interventions, care coordination and case management, and training of the care recipient with caregiver involvement. Notably, three studies incorporated culturally specific strategies in their training. Shata et al. (41) did not provide the relevant details. Mahdavi et al. (39) included prayers, discussions about divine issues based on the Quran. Zamani-Alavijeh et al. (15) suggested planning gender-specific activities, such as applying henna to female care recipients and attending poetry nights for male care recipients, as well as religious-related activities, including playing Quran recitation at the specific times of the day.

Various outcomes of the training programs were identified: burden and stress (*n* = 5) (28, 29, 41–43), depressive symptoms and anxiety (*n* = 4) (30, 36, 41, 43), subjective well-being (i.e., quality of life of caregivers) (*n* = 2) (29, 36), ability/knowledge (*n* = 3) (15, 40, 41), and physical health of the PLWD (*n* = 3) (36, 42, 43). Six studies evaluated outcomes on the family caregivers only (15, 28, 29, 30, 40, 41) while three evaluated outcomes of the dyad (36, 42, 43). There appear to be gaps in the psychosocial outcomes regarding social support and positive aspects of caregiving, and the outcomes primarily focused on the family caregivers’ mental health.

### Training for formal caregivers and the study outcomes in the MENA region

There were only two articles identified to report training for formal caregivers in long-term care facilities since 2014 (20, 44), reflecting inadequacy in evidence on dementia caregiving training for formal caregivers. The study designs employed were mixed methods (44) and one-group pre-posttest (20). It appears that there is a gap in a stringent quantitative design to rigorously evaluate the effectiveness of training programs for formal caregivers. Educational program was the only training identified for formal caregivers in the MENA region. Similar to educational programs for informal caregivers, topics focused on basic dementia disease knowledge, daily caregiving, and communication skills. In addition, Hayajneh and Shehadeh (44) focused on person-centered care; however, no culturally specific strategies were reported in the training in either of the included studies.

Two categories of study outcomes were identified: burden and stress (20), and ability and knowledge (44). Hayajneh and Shehadeh (44) evaluated the person-centered caregiving behaviors of formal caregivers quantitatively and employed semi-structured interviews to explore their experiences with the training program. There appear to be gaps in the psychosocial outcomes regarding depressive symptoms and anxiety, social support, and positive aspects of caregiving. Additionally, evaluation of PLWDs’ psychosocial health, such as neuropsychiatric symptoms and cognitive functions, was lacking.

## Discussion

This review provides evidence on training for informal and formal caregivers of PLWD and the study outcomes of the identified training in the MENA region. This is the first scoping review to map the evidence of training, based on primary research, for caregivers of PLWD in the MENA region. We used the PAGER framework to comprehensively describe and critique the findings of this scoping review, including gaps and research recommendations, which are discussed in the following section.

### Training and support for informal caregivers in the MENA region

The findings suggested that informal caregivers in the MENA region were mainly female (i.e., 65–85%) who were either the spouse or an adult child of PLWD, aligning with the global phenomenon. Most of the included studies reported a significant reduction in caregiver burden, depressive symptoms, and anxiety level (29, 30, 36, 37, 39, 42, 43). Additionally, significant improvements in caregiving knowledge and self-efficacy were demonstrated (15, 40, 41). These findings appear to address the caregiving challenges reported in previous literature (16) and may have enabled informal caregivers to provide relevant care and prepared them psychologically. Since multigenerational households are common in the MENA region and family ties among core and extended families are strong, they serve as a strength in the support system for caring for PLWD. Therefore, effective training in providing person-centered care to PLWD and psychological support to informal caregivers is paramount to help them remain in the caregiving role. However, it is difficult to conclude which training is most effective without a pooled effect size, which is beyond the scope of the current review. Therefore, future systematic reviews with meta-analyses are strongly recommended.

This review revealed evidence of training for informal caregivers of PLWD since 2005, with 12 interventional studies identified in the MENA region, including 10 psychosocial training programs. According to a systematic review on 131 randomized controlled trials, regarding evidence on psychosocial training for informal caregivers (11), North America contributed to the majority of research evidence (*n* = 53, 40.5%), followed by Europe (*n* = 42, 32.1%) and Asia (*n* = 23, 17.6%) while the MENA contributed to 3% (*n* = 4). There has been a moderate increase in the number of interventional studies on training informal caregivers of PLWD in the MENA region over the last 5 years. This is possibly due to the increased awareness of dementia care and the growing aging population worldwide.

Nevertheless, in general, there was inadequate evidence reported on the number and variety of training programs for informal caregivers of PLWD in the MENA region. This could be due to the understudying of dementia, attributed to stigmatization. Stigma and a lack of knowledge about dementia are barriers for PLWD to live fully with dignity and respect (45). According to a global awareness survey from Alzheimer’s Disease International (46), two-thirds of the participants (i.e., family caregivers and healthcare professionals) misbelieved that dementia is part of normal aging. The MENA region is no exception to dementia stigma; however, it may stem from cultural beliefs in fate, divine will, and spirits, which present a unique challenge (41). This underscores the need for culturally specific interventions to enhance public awareness.

In terms of research design, there appears to be a lack of qualitative research exploring informal caregivers’ experiences with training, which is essential to providing a comprehensive understanding of how and why the programs work. Such information will be critical for tailoring programs for the contextual and cultural needs of the MENA region. Tailoring content and format to caregivers’ experiences, preferences, and resources, and collaborating with informal caregivers, were suggested as important strategies for enhancing family caregivers’ active participation in psychosocial training (47). Therefore, a tailored training program may result in successful experiences, equipping informal caregivers with the relevant knowledge and skills for dementia caregiving.

Additionally, the training or support for informal caregivers in the MENA region appears to skew toward educational programs, according to the current findings. Globally, the WHO (1) recommends psychosocial training to equip informal caregivers with the knowledge and skills for dementia caregiving. In particular, psychoeducation that involved caregivers’ active participation was a feature highlighted by WHO (57). Psychoeducation, a type of psychosocial training that employs psychotherapeutic techniques such as cognitive-behavioral techniques, typically encompasses topics covered in educational programs and caregivers’ mental health and stress (11). Psychoeducation has been demonstrated to be effective in enhancing informal caregivers’ ability and knowledge in caregiving, burden and stress, depression, subjective well-being, and positive aspects of caregiving (11). Psychoeducation may be preferable to merely educational programs for addressing dementia knowledge and skills, as well as caregivers’ caregiving stress; however, it should be confirmed by further systematic reviews and meta-analyses. Additionally, only 2 psychoeducational programs were identified in the current review, highlighting a gap in their provision in the MENA region.

Current findings indicate a shortage of respite services, support groups, and care coordination in the MENA region. These psychosocial supports have demonstrated a positive effect on the subjective well-being of informal caregivers (e.g., affect and quality of life) (11), which are particularly important in the MENA region to facilitate social connections and peer support when most of the dementia caregiving falls on the shoulders of family members ((27–30)), who may have become socially isolated due to an intense caregiving schedule (48). Other types of psychosocial training may yield varying psychosocial outcomes to various degrees. For example, counselling and psychotherapy demonstrated the most considerable effect on reducing depression and anxiety (11). There is no one-size-fits-all solution. Therefore, it is recommended to offer a range of psychosocial training and support options to cater to the diverse needs of informal caregivers.

Moreover, study outcomes of the included training program for informal caregivers focused primarily on caregiver burden, stress and anxiety, and caregiving ability and knowledge. Positive aspects of caregiving, caregiving confidence, and social support were not evaluated. These psychosocial outcomes are important for reflecting the benefits of psychosocial training, shifting the perspective from deficit-based to strength-based (49, 50). Additionally, the use of positive measures is more likely to support active stakeholder engagement in developing, implementing, and evaluating validated interventions (51). A lack of diverse study outcomes may stem from the absence of validated questionnaires (13, 30). As such, translating and validating measurement tools suitable for Arab cultures is suggested as the future research direction in the MENA region.

### Training and support for formal caregivers in the MENA region

The findings revealed inadequate evidence on dementia caregiving training for formal caregivers in the MENA region, including the types of training, training content, research designs, study outcomes, and culturally specific strategies. A meta-review of 6 systematic reviews suggested that evidence of training and support for formal dementia caregivers came from the United Kingdom, the United States, and Asia (9). This highlights the gap in the provision of dementia caregiving training among formal caregivers in the MENA region. Without a well-prepared and adequately trained healthcare workforce, the region will face significant challenges in delivering equitable, effective, and compassionate care to PLWD.

The current review did not capture any evidence from clinical settings. This finding aligned with previous reviews (6, 9) and indicated that training for formal caregivers primarily focused on long-term care facilities worldwide. Of note, formal caregivers (i.e., domestic helpers, personal care aides, home health aides, and nurse assistants) are commonly recruited to provide dementia caregiving at home in some high-income regions such as the UAE. It is crucial to ensure these formal caregivers provide high-quality, relevant dementia care. This presents a unique challenge in the MENA region and underscores the opportunities to develop training for these types of formal caregivers.

Meanwhile, healthcare professionals’ training plays a significant role in fostering dementia caregiving competency needed to meet the needs in the MENA region. Indeed, related policies and training for professional caregivers regarding dementia care are already in place in some countries within the MENA region. For example, the UAE launched the National Strategy for Nursing and Midwifery—Roadmap for 2026 (52) to enhance the capacity, quality, and professionalism of nurses and midwives in delivering high-quality care that addresses societal needs. In line with this policy, the (53) provided healthcare workforce upskilling programs for existing family medicine doctors and general practitioners on dementia care. However, it is unclear whether trained personnel receive ongoing supervision, refresher training, or mentorship, and whether improvements in their behavior or clinical practices are sustained over time. Additionally, there is limited evidence on the long-term impact of dementia training, such as delaying institutionalization, improving the quality of life of PLWD, or reducing caregiver stress in this region.

To cultivate the right attitude, knowledge, and skills, undergraduate training for nurses, physicians, and allied health professionals in dementia diagnosis, management, and caregiving should not be overlooked. Higher institutions should offer structured, evidence-based dementia care training that integrates global best practices with the region’s unique socio-cultural and familial caregiving norms. The existing training programs vary widely in content, duration, target audience, accreditation, and modality. Some are short workshops; others are more formal diploma programs. There is no well-documented, unified curriculum for dementia care that spans the Emirates and integrates international best practices with the UAE’s cultural, linguistic, and religious contexts. Furthermore, while some programs are bilingual (English/Arabic), cultural adaptation beyond language (including nuanced norms of family caregiving, expectations, and stigma) is often under documented. It is therefore highly recommended to incorporate components of dementia knowledge and caregiving into the existing gerontology curriculum or to develop an independent gerontology subject that encompasses dementia-related knowledge and caregiving across the health professions curriculum in the UAE.

Notably, the UAE is progressively recognizing dementia as an integral part of the healthy aging agenda, and several policies and initiatives focusing on brain health and dementia prevention are being rolled out. Initiatives to promote brain health, including comprehensive healthcare, social, and psychological support, are in place to maintain older adults’ dignity and independence and to promote active community participation (54). Additionally, campaigns are in place to enhance public awareness of the early detection of functional, physiological, and cognitive decline in dementia. Moreover, upskilling programs for clinicians in primary care is underway for early screening and diagnoses. Details of these policies and initiatives are listed below.

1 National strategy for nursing and midwifery—roadmap for 2026

It is a key strategy to improve the capacity, quality, and professionalism of nurses and midwives in the UAE ((52)). The purpose of this initiative is to promote both public and specialist nursing academic programs, raise the quality of nursing care and midwifery services, and improve education, professional development, innovation, and research in these fields. Therefore, specialist training in dementia could be embedded in the curricula / professional development under this roadmap and therefore equipping frontline nurses with relevant knowledge and skills in dementia caregiving.

2 National framework for healthy aging 2025–2031 and “healthy aging” campaign

Launched by the MO HAP, this initiative features exclusive designs for individuals aged 60 and above and delivers comprehensive healthcare, social, and psychological support. It assists older adults in maintaining their dignity and independence, promoting active community participation (54). Additionally, early detection of functional, physiological, and cognitive changes associated with aging, which links directly to dementia detection, is the focus of this campaign.

3 Healthy aging and dementia response

MoHAP organized workshops, in collaboration with the WHO, to develop the “National Framework for Healthy Aging” and “Effective Health Response Plan for dementia (Alzheimer’s disease)” (54). This framework helps strengthen the collaboration between health and non-health sectors and to share best practices in dementia care.

4 Senior citizen and dementia-related services

Emirates Health Services has launched specialized mental health services at Al-Amal Psychiatric Hospital for senior citizens, including memory clinics and cognitive stimulation therapy (CST) (a non-pharmacological intervention) tailored to the UAE culture (55). The services include both inpatient and outpatient care, as well as recreational and social activities that aim to improve quality of life and mitigate memory disorders.

5 Healthcare workforce upskilling programs

The Department of Health of Abu Dhabi has launched the upskilling programs for health professionals (53). The program covers the topic “Dementia/Old Age Psychiatry” as part of its mental health priority topics. This endeavor aims to ensure that clinicians—especially in primary care—can screen, diagnose, manage simpler/stable cases, know when to refer, and provide more holistic care that includes mental health for older adults.

6 Workforce planning and strategy development

MoHAP has convened experts and stakeholders to discuss a long-term strategy for the national health workforce, to align with changing health demands. This includes identifying required competencies, preparing for evolving healthcare needs (which presumably include the rising prevalence of dementia), and ensuring training and education systems are up to date (56). In particular, the Department of Health’s “Tawteen” initiative aims to increase the proportion of UAE nationals in the healthcare workforce, thereby requiring training and capacity-building for dementia care (56).

### Recommendations

While the above efforts show promise; however, significant gaps remain, including inadequate training structures, limited content depth, inconsistent delivery, insufficient reach across healthcare roles, and a lack of standardized evaluation methods. The first is a clear policy framework for dementia training to support such programs in the UAE, both for health professionals and caregivers. Another drawback is the standardization of curricula and competencies for dementia, which integrates international best practices with the UAE’s cultural, linguistic, and religious contexts.

While policy momentum is strong in the UAE, translating it into widespread, evaluated professional training and standardized competency frameworks is still in development. The national policy and workshops indicate recognition, but what is urgently needed is operationalization—well-funded, regularly delivered training across all emirates, embedded into the training, accreditation, and licensing of healthcare professions. It is unclear whether trained personnel in the UAE receive ongoing supervision, refresher training, or mentorship, and whether improvements in their behavior or clinical practices are sustained over time. Additionally, there is limited UAE-specific evidence on the long-term impact of dementia training—such as delaying institutionalization, improving quality of life, or reducing caregiver stress.

Moreover, most of these programs are located in major emirates (Dubai, Abu Dhabi, Sharjah, Ajman). Rural or remote areas, smaller emirates may have less access. Also, informal caregivers (family members) are often not formally included in many training programs. Cultural norms in the UAE—such as strong expectations of family caregiving, stigma surrounding dementia, and reluctance to seek institutional or external support—can affect both the uptake of caregiver training and the relevance of its content. While these cultural factors are occasionally acknowledged, they are rarely fully integrated into the design or evaluation of training programs.

### Implications for nursing practice and research

Further research on systematic reviews to evaluate overall training effectiveness is warranted. Additionally, more research using quantitative and qualitative designs is necessary to provide evidence on various types of training and support for informal and formal caregivers of PLWD. Studies evaluating culturally relevant psychosocial interventions are highly recommended. Moreover, evaluating study outcomes from a strength-based perspective, such as the positive aspects of caregiving, caregiving confidence, and social support, may help foster stakeholders’ active engagement in developing and implementing training. Furthermore, validating more region-specific measurement tools that align with Arab culture may facilitate the evaluation of training and support in the MENA region. Last but not least, raising public awareness of dementia, addressing its stigma, and preparing a competent workforce on dementia care are the way forward in this region.

### Limitations

Although an extensive search of the literature was conducted, this review only included works items published in English and those published in peer-reviewed journals. Relevant articles published in other languages were omitted. Most of the included studies were quantitative, with only one mixed-methods study, and no qualitative study was identified.

## Conclusion

This scoping review mapped the evidence of dementia caregiving training for informal and formal caregivers in the MENA region. We employed the PAGER framework to analyse the findings, identifying advances and gaps, discussing evidence for practice, and suggesting recommendations for practice and research. A scarcity of dementia caregiving training for informal and formal caregivers in the MENA region was revealed. Training for caregivers skewed toward educational programs with evaluations focused on a deficit-based perspective that primarily focused on the caregivers. Further research on systematic reviews to evaluate overall training effectiveness, validation of region-specific measurement tools, and culturally specific interventional studies with both quantitative and qualitative evaluations of various types of psychosocial training on strength-based outcomes is highly recommended.

## Data Availability

The original contributions presented in the study are included in the article/supplementary material, further inquiries can be directed to the corresponding author.

## References

[1] WHO (2025). Dementia: key facts. Available online at: https://www.who.int/news-room/fact-sheets/detail/dementia#:~:text=Alzheimer%20disease%20is%20the%20most,dependency%20among%20older%20people%20globally (Accessed September 4, 2025).

[2] Arthurton L Barbarino P Anderson R Schlaepfer B Salehi N Knapp M. Dementia is a neglected noncommunicable disease and leading cause of death. Nat Rev Neurol. (2025) 21:63–4. doi: 10.1038/s41582-024-01051-w, 39800817

[3] GBD 2019 Dementia Forecasting Collaborators. Estimation of the global prevalence of dementia in 2019 and forecasted prevalence in 2050: an analysis for the global burden of disease study 2019. Lancet Public Health. (2022) 7:e105–25. doi: 10.1016/S2468-2667(22)00294-8, 34998485 PMC8810394

[4] Alsaadi T Almadani A AlRukn S AlRukn S Hassan A Sarathchandran P . Expert guidance on cognitive impairment in Alzheimer's disease: a practical seven-step approach from the United Arab Emirates. Neurol Ther. (2025) 14:2507–35. doi: 10.1007/s40120-025-00833-8, 41045350 PMC12623560

[5] PR Newswire (2023). Eisai’s approach to U.S. pricing for Leqembi™ (lecanemab), a treatment for early Alzheimer’s disease, sets forth our concept of ‘societal value of medicine’ in relation to ‘price of medicine. Available online at: https://media-us.eisai.com/2023-01-06-EISAIS-APPROACH-TO-U-S-PRICING-FOR-LEQEMBI-TM-LECANEMAB-,-A-TREATMENT-FOR-EARLY-ALZHEIMERS-DISEASE,-SETS-FORTH-OUR-CONCEPT-OF-SOCIETAL-VALUE-OF-MEDICINE-IN-RELATION-TO-PRICE-OF-MEDICINE#:~:text=While%20we%20estimate%20the%20per,U.S.%20patient%20weight%20of%2075kg (Accessed November 11, 2025).

[6] Lee KH Lee JY Kim B. Person-centered Care in Persons Living with Dementia: a systematic review and Meta-analysis. Gerontologist. (2022) 62:e253–64. doi: 10.1093/geront/gnaa207 33326573 PMC9019632

[7] Ahmad MA Kareem O Khushtar M Akbar M Haque MR Iqubal A . Neuroinflammation: a potential risk for dementia. Int J Mol Sci. (2022) 23:616. doi: 10.3390/ijms23020616, 35054805 PMC8775769

[8] Long S Benoist C Weidner W. World Alzheimer report 2023: Reducing dementia risk: Never too early, never too late. London: Alzheimer’s Disease International (2023).

[9] Sefcik JS Boltz M Dellapina M Gitlin LN. Are interventions for formal caregivers effective for improving dementia care? A systematic review of systematic reviews. Innov Aging. (2022) 6:igac005. doi: 10.1093/geroni/igac005 35496650 PMC9042653

[10] Kitwood T. Dementia reconsidered: The person comes first. Philadelphia: Open University Press (1997).

[11] Cheng ST Li KK Losada A Zhang F Au A Thompson LW . The effectiveness of nonpharmacological interventions for informal dementia caregivers: an updated systematic review and meta-anaylsis. Psychol Aging. (2020) 35:55–77. doi: 10.1037/pag0000401 31985249

[12] Walter E Pinquart M. How effective are dementia caregiver interventions? An updated comprehensive meta-analysis. Gerontologist. (2020) 60:e609-e619. doi: 10.1093/geront/gnz118 33226434

[13] Cheng ST Au A Losada A Thompson LW Gallagher-Thompson D. Psychological interventions for dementia caregivers: what we have achieved, what we have learned. Curr Psychiatry Rep. (2019) 21:59. doi: 10.1007/s11920-019-1045-9 31172302 PMC6554248

[14] Kane T Hammad SH Islam N Al-Wattary N Clark J Daher-Nashif S. Dementia caregiving in the Middle East and North Africa: a scoping review. Transcult Psychiatry. (2021) 58:844–58. doi: 10.1177/13634615211036404, 34407707

[15] Zamani-Alavijeh F Zahed S Emami M Bazargan-Hejazi S Barekatain M Hassanzadeh A . The effect of educational intervention based on the progressively lowered stress threshold extended model on the process of caregiving for people with dementia. Psychogeriatrics. (2023) 23:1019–26. doi: 10.1111/psyg.13022, 37679996

[16] Hussein S Ismail M. Ageing and elderly care in the Arab region: policy challenges and opportunities. Ageing Int. (2017) 42:274–89. doi: 10.1007/s12126-016-9244-8, 28890585 PMC5569126

[17] Stall NM Kim SJ Hardacre KA Shah PS Straus SE Bronskill SE . Association of informal caregiver distress with health outcomes of community-dwelling dementia care recipients: a systematic review. J Am Geriatr Soc. (2019) 67:609–17. doi: 10.1111/jgs.15690 30536383

[18] Bar-Tur L. Fraught triads – treating older women in crisis living with a migrant live-in caregiver and frail husband. Educ Gerontol. (2024) 50:944–54. doi: 10.1080/03601277.2024.2366078

[19] Dwolatzky T Brodsky J Azaiza F Clarfield M Jacobs JM Litwin H. Coming of age: health-care challenges of an ageing population in Israel. Lancet. (2017) 389:2542–50. doi: 10.1016/s0140-6736(17)30789-4, 28495114

[20] Rezq KA Gutierrez JV. Effect of dementia educational program on formal caregivers burden in elderly homes. J Pharm Res Int. (2021) 33:15–23. doi: 10.9734/jpri/2021/v33i54A33714

[21] Paul P Mahfoud ZR Malik RA Kaul R Muffuh NP Al-Sheikhly DCA. Knowledge, awareness, and attitude of healthcare stakeholders on Alzheimer's disease and dementia in Qatar. Int J Environ Res Public Health. (2023) 20:4535. doi: 10.3390/ijerph20054535 36901551 PMC10002196

[22] Samir AA Hageen AW Elbarbary K Elamir AH Abdel-Fattah MA Alameldin MM . Assessing Alzheimer's disease knowledge among Egyptian medical students in the context of recent educational reforms. BMC Med Educ. (2025) 25:654. doi: 10.1186/s12909-025-07258-9, 40325427 PMC12054285

[23] Gkioka M Schneider J Kruse A Tsolaki M Moraitou D Teichmann B. Evaluation and effectiveness of dementia staff training programs in general hospital settings: a narrative synthesis with Holton's three-level model applied. J Alzheimer's Dis. (2020) 78:1089–108. doi: 10.3233/JAD-200741 33104033 PMC7739966

[24] Oh E Moon S Chung D Choi R Hong GS. The moderating effect of care time on care-related characteristics and caregiver burden: differences between formal and informal caregivers of dependent older adults. Front Public Health. (2024) 12:1354263. doi: 10.3389/fpubh.2024.1354263, 38638476 PMC11024244

[25] Turner A Eccles FJ Elvish R Simpson J Keady J. The experience of caring for patients with dementia within a general hospital setting: a meta-synthesis of the qualitative literature. Aging Ment Health. (2017) 21:66–76. doi: 10.1080/13607863.2015.1109057 26553275

[26] Diaz-Gil A Brooke J Kozlowska O Pendlebury S Jackson D. Care needs of people with dementia in the peri-operative environment: a systematic review. Dementia (London). (2020) 19:1889–906. doi: 10.1177/1471301218809225, 30419182

[27] Abdelhalim DSA Ahmed MM Hussein HA Sarhan MD Khalaf OO. A skill-based multimodal intervention for dementia caregivers: impact on burden and anxiety. Aging Clin Exp Res. (2025) 37:95. doi: 10.1007/s40520-025-02985-x 40095192 PMC11914238

[28] Jahani L Abolhassani S Babaee S Omranifard V. Effects of a compassion-based program on the grief experienced by caregivers of people suffering from dementia: a randomized controlled clinical trial. BMC Nurs. (2022) 21:198. doi: 10.1186/s12912-022-00980-5, 35879751 PMC9316726

[29] Tawfik NM Sabry NA Darwish H Mowafy M Soliman SSA. Psychoeducational program for the family member caregivers of people with dementia to reduce perceived burden and increase patient’s quality of life: a randomized controlled trial. J Prim Care Community Health. (2021) 12:1–7. doi: 10.1177/21501327211014088 PMC8120530 33971764

[30] Werner P Clay OJ Goldstein D Kermel-Schifmann I Herz MK Epstein C . Assessing an evidence-based intervention for spouse caregivers of persons with Alzheimer’s disease: results of a community implementation of the NYUCI in Israel. Aging Ment Health. (2021) 25:1676–83. doi: 10.1080/13607863.2020.1774740 32496814

[31] Peters MDJ Godfrey C Mc Inerney P Munn Z Tricco AC Khalil H. "Chapter 11: scoping reviews" In: Aromataris E Munn Z, editors. JBI manual for evidence synthesis: JBI (2020). Available online at: Available from: https://synthesismanual.jbi.global (Accessed September 10, 2025).

[32] Bradbury-Jones C Aveyard H Herber OR Isham L Taylor J O'Malley L. Scoping reviews: the PAGER framework for improving the quality of reporting. Int J Soc Res Methodol. (2022) 25:457–70. doi: 10.1080/13645579.2021.1899596

[33] Tricco AC Lillie E Zarin W. PRISMA extension for scoping reviews (PRISMA-ScR): checklist and explanation. Ann Intern Med. (2018) 169:467–73. doi: 10.7326/M18-0850, 30178033

[34] United Nations. (2021). Middle east and North Africa section-profile. Available online at: https://www.ohchr.org/en/countries/middle-east-north-africa-region/middle-east-north-africa-section-hq (accessed on September 10, 2025).

[35] Arksey H O’Malley L. Scoping studies: towards a methodological framework. Int J Soc Res Methodol. (2005) 8:19–32. doi: 10.1080/1364557032000119616

[36] Kuzu N Beşer N Zencir M Sahiner T Nesrin E Ahmet E . Effects of a comprehensive educational program on quality of life and emotional issues of dementia patient caregivers. Geriatr Nurs. (2005) 26:378–86. doi: 10.1016/j.gerinurse.2005.09.015, 16373183

[37] Pahlavanzadeh S Heidari FG Maghsudi J Ghazavi Z Samandari S. The effects of family education program on the caregiver burden of families of elderly with dementia disorders. Iran J Nurs Midwifery Res. (2010) 15:102–8. 21589771 PMC3093163

[38] Heydari M Razban F Mirzaei T Heidari S. The effect of problem oriented coping strategies training on quality of life of family caregivers of elderly with Alzheimer. Asian J Nurs Educ Res. (2017) 7:168. doi: 10.5958/2349-2996.2017.00034.9

[39] Mahdavi B Fallahi-Khoshknab M Mohammadi F Hosseini MA Haghi M. Effects of spiritual group therapy on caregiver strain in home caregivers of the elderly with Alzheimer's disease. Arch Psychiatr Nurs. (2017) 31:269–73. doi: 10.1016/j.apnu.2016.12.003, 28499566

[40] Salamizadeh A Mirzaei T Ravari A. The impact of spiritual care education on the self-efficacy of the family caregivers of elderly people with Alzheimer’s disease. Int J Commun Based Nurs Midwifery. (2017) 5:231–8. PMC5478743 28670585

[41] Shata ZN Amin MR El-Kady HM Abu-Nazel MW. Efficacy of a multi-component psychosocial intervention program for caregivers of persons living with neurocognitive disorders, Alexandria, Egypt: a randomized controlled trial. Avicenna J Med. (2021) 7:54–63. doi: 10.4103/2231-0770.203610 PMC5398004 28469987

[42] Javadpour A Ahmadzadeh L Bahredar MJ. An educative support group for female family caregivers: impact on caregivers psychological distress and patient's neuropsychiatry symptoms. Int J Geriatr Psychiatry. (2009) 24:469–71. doi: 10.1002/gps.2138 18937279

[43] Abdelhalimet N Knezevic B Sousa P Tella S Srulovici E Strametz R . Defining informal caregivers by their characteristics safety roles and training needs in Europe. Sci Rep. (2025) 15:24375. doi: 10.1038/s41598-025-08540-y 40628820 PMC12238542

[44] Hayajneh FA Shehadeh A. The impact of adopting person-centred care approach for people with Alzheimer's on professional caregivers' burden: an interventional study. Int J Nurs Pract. (2014) 20:438–45. doi: 10.1111/ijn.12251, 25039325

[45] The Lancet Neurology. Dementia-related stigma is still pervasive. Lancet Neurol. (2024) 23:1063. doi: 10.1016/S1474-4422(24)00404-6 39424545

[46] Alzheimer’s Disease International. World Alzheimer report 2024: Global changes in attitudes to dementia. London: Alzheimer’s Disease International (2024).

[47] Chan HM Ho KHM Pang RCK Chan HYL. Strategies and factors to enhance active participation of family caregivers of people with dementia in psychoeducation: a scoping review. Dementia. (2023) 23:272–91. doi: 10.1177/14713012231220231, 38091474

[48] Samia LW O’Sullivan A Fallon KC Aboueissa AM Hepburn KW. Building on self-efficacy for experienced family caregivers: the savvy advanced program. The Gerontologist. (2019) 59:973–82. doi: 10.1093/geront/gny016 29546325

[49] Gaugler JE Bain LJ Mitchell L Finlay J Fazio S Jutkowitz E . Reconsidering frameworks of Alzheimer’s dementia when assessing psychosocial outcomes. Alzheimers Dement. (2019) 5:388–97. doi: 10.1016/j.trci.2019.02.008, 31463361 PMC6708985

[50] Kolanowski A Behrens L Lehman E Oravecz Z Resnick B Boltz M . Living well with dementia: factors associated with nursing home residents’ affect balance. Res Gerontol Nurs. (2020) 13:21–30. doi: 10.3928/19404921-20190823-01, 31454406 PMC6980972

[51] Fulmer T Mate KS Berman A. The age-friendly health system imperative. J Am Geriatr Soc. (2018) 66:22–4. doi: 10.1111/jgs.15076 28876455

[52] Ministry of Health & Protection (2022). UAE National Strategy for nursing/midwifery: a roadmap to 2026. Available online at: https://mohap.gov.ae/documents/d/guest/national-strategy-for-nursing-and-midwifery-en-pdf-1#:~:text=The%20UAE%20National%20Strategy%20for%20Nursing%2FMidwifery%3A%20A%20Roadmap%202026,support%20and%20quality%20standards%20implementation (accessed on September 10, 2025).

[53] Department of Health. (2025a). Healthcare workforce upskilling Programmes, Abu Dhabi. Available online at: https://www.doh.gov.ae/en/programs-initiatives/healthcare-workforce-upskilling-programs?utm_source=chatgpt.com (Accessed September 10, 2025).

[54] News Bureau (2025). Healthy ageing 2025–2031: MoHAP launches elderly care drive. September 25. Emirati Times. Available online at: https://emiratitimes.com/mohap-healthy-ageing-framework/?utm_source=chatgpt.com (accessed on September 10, 2025).

[55] Emirates Health Services. (2024). Emirates health services streamlines services and delivers pioneering Care for Senior Citizens, United Arab Emirates. Available online at: https://www.ehs.gov.ae/en/media-center/news/emirates-health-services-streamlines-services-and-delivers-pioneering-care-for-senior-citizens?utm_source=chatgpt.com (Accessed September 10, 2025).

[56] Department of Health. (2025b). Tawteen and the sustainability of the healthcare workforce, Abu Dhabi. Available online at: https://www.doh.gov.ae/en/programs-initiatives/tawteen?utm_source=chatgpt.com (Accessed September 10, 2025).

[57] World Health Organization (2012). Interventions for carers of people with dementia. Available online at: https://www.who.int/docs/default-source/mentalhealth/mhgap/dementia/dementia-q9.pdf?sfvrsn=d21cb3f9_0 (Accessed September 10, 2025).

